# Supplementary material for: Photocatalytic degradation of methylene blue under natural sunlight using iron titanate nanoparticles prepared by a modified sol–gel method
Source: R Soc Open Sci. 2020 Sep 2;7(9):200708. doi: 10.1098/rsos.200708 (PMC7540765; doi:10.1098/rsos.200708)
Supplement: Figure 1;Figure 2;Figure 3 [file rsos200708supp1.docx]

Fig. S1 Time dependent changes in absorption spectra of MB dye aqueous solution (100 mg L^-1^) in presence of Fe_2_TiO_5_ photocatalyst (100mg) under sunlight irradiation

Fig. S2 Effect of the initial dye concentration


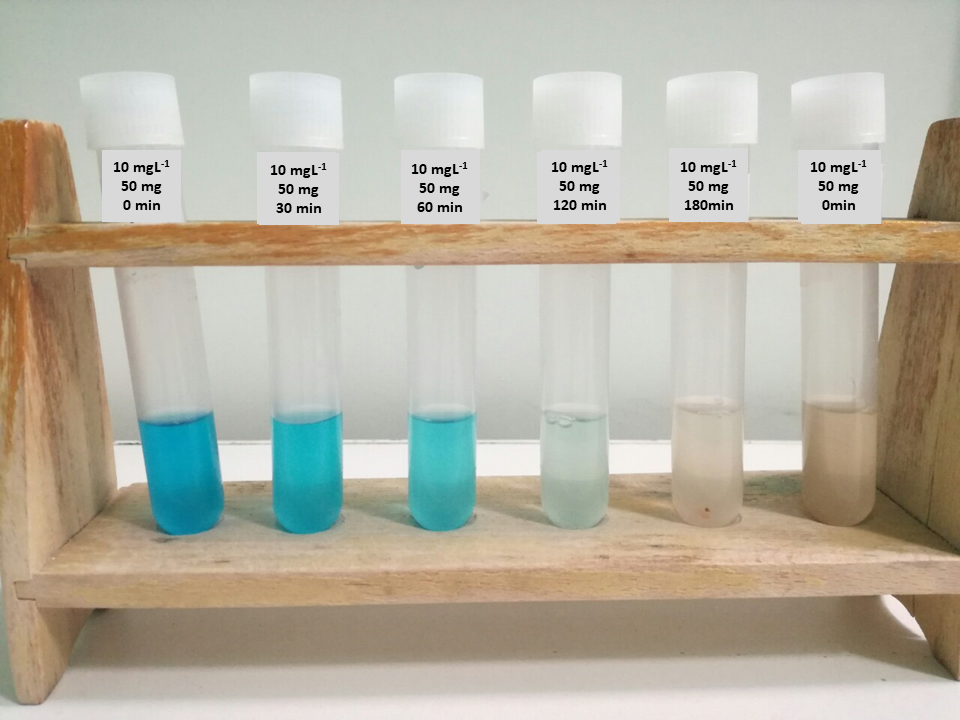


Fig S3. Visual observation of colour changing from blue to colourless (pH=11, MB=10 mg L^-1^, Fe_2_TiO_5_=50mg)
